# Supplementary material for: Population-scale sequencing resolves determinants of persistent EBV DNA
Source: Nature. 2026 Jan 28;650(8102):664–72. doi: 10.1038/s41586-025-10020-2 (PMC12888827; doi:10.1038/s41586-025-10020-2)
Supplement: Supplementary file 1 — Supplementary Notes 1–6 and Figs. 1–4. [file 41586_2025_10020_MOESM1_ESM.docx]

**Supplemental Information for**

# **Population-scale sequencing resolves determinants of persistent EBV DNA**

**Table of contents**

**Supplemental Note 1. Multi-modal characterization of EBV in peripheral blood.**

**Supplemental Note 2. Characterizing persistence of EBV DNA in saliva.**

**Supplemental Note 3. EBV DNAemia is a biomarker of complex traits.**

**Supplemental Note 4. Supporting analyses for UKB NFE genetic associations.**

**Supplemental Note 5. Meta-analysis of UKB EBV DNAemia GWAS with diverse ancestries.**

**Supplemental Note 6. Genetic diversity in EBV genomes across UKB and AoU.**

**Supplementary Figures**

**Supplementary Fig. 1. Characterization of EBV latency in large genomics datasets.**

**Supplementary Fig. 2. Quantification of EBV DNA from AoU saliva WGS samples.**

**Supplementary Fig. 3. Supporting analyses for NFE genetic association studies.**

**Supplementary Fig. 4. Additional genetic association studies.**

**References**

**Supplemental Note 1. Multi-modal characterization of EBV in peripheral blood.**

To better characterize the source of the detected EBV DNA, we analyzed additional population-scale data from cohorts of primarily healthy participants to distinguish between lytic and latent viral states. In memory B cells, EBV predominantly remains in a transcriptional state (termed latency 0) with a limited viral gene expression program[^1^](https://paperpile.com/c/FN0JXJ/dITGP). In contrast, viruses in lytic reactivation express a wide range of viral proteins that enable immune evasion[^1^](https://paperpile.com/c/FN0JXJ/dITGP). As UKB and AoU lack concomitant RNA-seq data, we assessed peripheral blood EBV levels from matched WGS and RNA-seq from 681 Genotype-Tissue Expression Project donors (GTEx, **Supplementary Fig. 1a**). After correcting for contig biases as in UKB and AoU, we observed detectable EBV DNA in 24.8% of individuals, but EBV RNA in only 0.44% (3 donors total). Each of the three donors had only a single EBV-derived read, and only one of these donors also had corresponding EBV DNA detected (**Supplementary Fig. 1b,c; Methods**). These findings indicate that EBV DNA presence in peripheral blood is overwhelmingly not accompanied by detectable viral RNA expression, supporting that the viral DNA detected in UKB and AoU likely reflects latent carriage rather than active replication. Paired analyses of EBV DNAemia and RNA-seq profiles showed elevated estimated B cell proportions and lower B cell activation states for individuals with EBV DNAemia, reflecting that peripheral blood cell composition may mediate viral DNA persistence (**Supplementary Fig. 1d,e**).

To corroborate this observation of minimal viral gene expression, we reanalyzed the OneK1K cohort, which profiled approximately 1.4 million peripheral blood mononuclear cells (PBMCs) from nearly 1,000 healthy donors[^2^](https://paperpile.com/c/FN0JXJ/9eGFz). Analyzing over 53.9 billion reads, we observed only 1 EBV transcript across all donors and all cells from this cohort (**Supplementary Fig. 1f,g**). To confirm EBV transcript detection sensitivity with scRNA-seq, we reanalyzed scRNA profiles of LCL, in which EBV expresses a latency III program[^3^](https://paperpile.com/c/FN0JXJ/AhPS1), and kidney transplant[^4^](https://paperpile.com/c/FN0JXJ/Ifq5h) samples assayed with the same sequencing workflow as OneK1K. In both RNA-seq datasets, we detected 100s-1,000s of EBV unique molecular identifiers (UMIs; indicating individual EBV RNA molecules), including rare cells with high transcriptional activity (consistent with HHV-6[^5^](https://paperpile.com/c/FN0JXJ/Rw7q3); **Supplementary Fig. 1h,i**).

Together, the analyses of the GTEx and OneK1K datasets suggest that EBV is transcriptionally silent at the time of blood sample collection for most healthy individuals. As latently infected cells have little to no viral gene expression[^6^](https://paperpile.com/c/FN0JXJ/u40N2), our analyses support the idea that at any given time, healthy adults are unlikely to have active EBV lytic replication or reactivation[^7^](https://paperpile.com/c/FN0JXJ/CFhk9), which occur sporadically over a lifetime[^1^](https://paperpile.com/c/FN0JXJ/dITGP). We emphasize that recent sensitive methods specific for EBV have detected very low amounts of EBV transcripts in peripheral blood B cells of healthy donors[^8^](https://paperpile.com/c/FN0JXJ/alQr). The failure to detect EBV transcripts in the analyzed RNA-seq datasets may reflect that previous methods are not sensitive enough to capture low levels of EBV transcripts, if present, and further work is needed to fully determine the latency state of the virus. We note, however, that in the same paper, the 10 healthy controls had 0-3 EBV-infected B cells detected per 10,000 cells, and no subpopulation of B cells with EBV lytic reactivation gene expression was detected[^8^](https://paperpile.com/c/FN0JXJ/alQr), corroborating our observations of largely latent EBV in the blood of healthy individuals.

The EBV DNA levels we detected were from a single snapshot in time (i.e., the time of blood collection for WGS). To further characterize the dynamics of EBV persistence from UKB donors, we inspected longitudinal serology measurements from an available subset of individuals. Here, 262 individuals, including 17 individuals with EBV DNAemia, had baseline serology profiles and WGS, with additional serology measurements taken 2–6 years following the initial sample collection[^9^](https://paperpile.com/c/FN0JXJ/vlc7a) (**Methods**). We analyzed the change in titers against all four EBV antigens, finding no significant difference in longitudinal fold changes of antibody titers when stratified by EBV DNAemia (**Supplementary Fig. 1j,k**). Additionally, we observed that 0 individuals with EBV DNAemia had seroconversion or seroreversion over longitudinal samples for any of the four antigens. The lack of resolution in antibody isotypes means that seropositivity does not necessarily indicate ongoing or recent reactivation (**Methods**), and we observed no evidence of variable antibody responses that would result from recent reactivation or infection in this subset of EBV DNAemia individuals. In sum, while a small proportion of the 735,954 donors in UKB and AoU were likely experiencing lytic infection or reactivation during blood sampling, these analyses suggest that EBV DNA captured by WGS of population biobanks likely reflects latent infection for most individuals. Prior studies in immunosuppressed populations indicate that EBV DNAemia can be attributed to the abundance of latently infected B cells in a sample[^7^](https://paperpile.com/c/FN0JXJ/CFhk9), which we anticipate drives our detection of EBV DNA across our cohorts.

**Methods***EBV DNA and RNA detection in GTEx*

For reanalysis of EBV reads from GTEx, we obtained the mapped alignment files (.bam) from WGS and RNA-seq of PBMCs for 681 donors, in which both were profiled for each individual. The GTEx consortium workflow hosts reads aligned to the hg38 reference, which included chrEBV (verified to be in all libraries). We downloaded .bam files from Anvil after obtaining permissions through dbGaP and subsequently extracted any reads mapping to the chrEBV contig for all sequencing libraries. We then filtered for paired-end reads with a map alignment quality exceeding 30 that did not overlap the annotated repetitive regions (**Supplementary Fig. 1a,b**), aggregated the per-library read abundance, and paired the RNA and DNA samples using the master GTEx metadata file. A total of 3 donors had detectable EBV RNA, all of whom had only one read, whereas 178 WGS donors had detectable EBV DNA, including one donor that had 761 reads, corresponding roughly to a copy number of 1 EBV genome per 20 cells (**Supplementary Fig. 1c**). Of the three individuals with detectable EBV RNA, only 1 had detectable EBV DNA. Further analyses of the GTEx RNA-seq data included an estimation of cell type composition using CIBERSORT[^10^](https://paperpile.com/c/FN0JXJ/WE0yZ), and the B cell activation module was estimated using the AddModuleScore from Seurat[^11^](https://paperpile.com/c/FN0JXJ/eJYIa) of the B cell activation genes in the PANTHER database.

*Multimodal analyses of EBV in peripheral blood*

As an independent dataset to examine whether EBV is latent (rather than an active infection), we quantified scRNA-seq data of PBMCs from ~1,000 individuals profiled via single-cell sequencing[^2^](https://paperpile.com/c/FN0JXJ/9eGFz) using kallisto[^12^](https://paperpile.com/c/FN0JXJ/NEjIG) for mRNA quantification (OneK1K cohort; analogous to our previous execution with HHV-6[^5^](https://paperpile.com/c/FN0JXJ/Rw7q3)). After verifying that non-human reads were provided as part of the upload (i.e., ~90-95% of the reads mapped to the hg38 reference), we reanalyzed a total of 53,872,337,003 paired-end sequencing reads from this consortium, identifying only 1 UMI that was classified as uniquely mapping to the EBV transcriptome (the BARF0 EBV gene). To control for the possibility that EBV is poorly detected by 10x scRNA-seq, we reanalyzed scRNA profiles of LCL[^3^](https://paperpile.com/c/FN0JXJ/AhPS1) and kidney transplant[^4^](https://paperpile.com/c/FN0JXJ/Ifq5h) samples assayed with the same sequencing workflow as OneK1K using the same kallisto workflow. In both datasets, we detected 100s-1,000s of unique molecular identifiers (UMIs) derived from the EBV transcriptome, including rare cells with 10+ viral UMIs from both datasets. These analyses confirmed that there were no obvious technical limitations of EBV detection with the scRNA-seq workflow, and that our limited detection of viral UMIs could be interpreted as true lack of viral expression.

As an additional measure of assessing EBV latency, we considered the subset of individuals in UKB for whom longitudinal serology measurements were available. Of the 277 individuals with longitudinal serology, 262 had: a) WGS profiled from the baseline sample (“instance 0” in UKB data field 32056); b) serology assessed at the baseline sample; and c) a replicate serology measure from a separate blood draw taken 2–6 years following the baseline[^9^](https://paperpile.com/c/FN0JXJ/vlc7a) (note: the precise dates were not available). There were at most two time points available for serology measurements, and no longitudinal WGS samples were available in either biobank. We analyzed the log_2_ fold change in titers against all 4 EBV antigens for these 262 individuals, observing no significant difference (**Supplementary Fig. 1j,k**). Moreover, we observed that 0 individuals with EBV DNAemia had seroconversion or seroreversion over longitudinal samples for any of the four antigens, using the seropositivity definitions for each antigen as provided by the UKB reference panel (VCA p18 > 250; EBNA-1 > 150; ZEBRA > 100; EA-D > 100, units: MFI)[^13^](https://paperpile.com/c/FN0JXJ/SS3pU). We note that while serology can be used to distinguish between active, latent, and recently reactivated EBV states[^13^](https://paperpile.com/c/FN0JXJ/SS3pU), the UKB serology measurements do not provide the detailed isotype information needed to fully distinguish between these states in the individuals measured.

**Supplemental Note 2. Characterizing persistence of EBV DNA in saliva.**

In addition to establishing latency predominantly in peripheral memory B cells, EBV can be periodically released from chronically infected pharyngeal epithelial cells, including from the same individuals with latent infection in B cells[^14^](https://paperpile.com/c/FN0JXJ/3QqAy). For all donors in the UK Biobank (UKB) and all donors in All of Us (AoU) through version 7 of the data release, peripheral blood was the input material for whole genome sequencing (WGS). In addition, for ~50,000 individuals released in version 8 of AoU, saliva was used as the input material for WGS, which provided an opportunity to study EBV DNAemia from this distinct anatomic site. The individuals with WGS libraries from saliva did not overlap any of the blood-derived profiles, so direct comparisons were not possible. However, this dual data source enabled the study of a distinct reservoir of EBV that aided in contextualizing our results focused on blood in the main text.

48,899 individuals were profiled via WGS using saliva DNA as input material that were made available in the All of Us CDRv8 genomic data release. Reads aligning to chrEBV were extracted via the same method as described for peripheral blood (**Supplementary Fig. 2a**). When examining coverage across the EBV genome, we note that there were no marked regions of abnormally high coverage as seen in the blood (**Supplementary Fig. 2b**). This observation is similar to the EBV DNA quantification in WGS performed on LCLs in the demo notebook provided in the GitHub repository[^15^](https://paperpile.com/c/FN0JXJ/g1iml). We reasoned that this is due to the extremely high loads of EBV DNA that drown out the non-specific signal from the human genome.

To facilitate comparisons between blood and saliva EBV DNA quantification, we mirrored the analyses done in the main manuscript for blood (**Supplementary Fig. 2c-e**). We observed that 20.1% of individuals with detectable EBV DNA in saliva only had reads aligned to the biased regions in the main manuscript (**Fig. 1b**), and after masking the biased regions, 29,353 individuals (60%) had detectable EBV DNA. Notably, in addition to the much higher detection rates for saliva, the levels of detectable EBV DNA were several orders of magnitude higher in saliva compared to blood (**Supplementary Fig. 2d**). We interpret this to mean that, if there exists a high level of EBV DNA reads in a WGS sample, our approach can readily detect and quantify this molecular feature. Combined with our simulations in **Extended Data Fig. 1**, for our peripheral blood samples, we estimate that sequencing ~100x deeper per-whole genome would detect EBV in ~90% of individuals. Using the same 1.2 EBV genomes per 10^4^ cells threshold as blood, there were 24,911 individuals (50.9%) with EBV DNAemia in saliva. To characterize these individuals, we plotted EBV DNAemia rates stratified by age and sex at birth. We observed a male-higher bias, but no obvious association with age (**Supplementary Fig. 2e**). To assess the genetic basis of saliva EBV DNAemia, we assessed three different phenotypes for genome-wide association studies using individuals of European ancestry (**Methods**). In contrast to the 22 significant loci identified in the blood-based EBV DNAemia GWAS (**Fig. 3a**), the only significant locus identified in all three models was in the MHC locus (**Supplementary Fig. 2f**). This disparity of non-MHC, genome-wide significant results emphasize the distinct polygenic architecture of EBV DNAemia in peripheral blood compared to saliva, suggesting a distinct immune basis that underlies the blood-based EBV DNAemia, the focus of our work. Despite the lack of non-MHC loci, we note that associations of four-digit HLA alleles were consistent for both MHC class I and II (**Supplementary Fig. 2g,h**).

These analyses provide extra context for our results on peripheral blood-derived EBV DNA. First, the overall framework of identifying EBV DNA reads from existing WGS libraries will scale to other input materials and allow for future study of other biological reservoirs or other viral infections. Second, EBV DNA detection is qualitatively and quantitatively different in peripheral blood than saliva, as we see substantially more donors with detectable EBV than when assaying saliva (**Supplementary Fig. 2d**). This likely reflects the distinct biological dynamics of the infection, reactivation, and shedding of EBV in the oropharynx compared to peripheral blood. Finally, despite performing genetic association mapping in three different ways for saliva, we observed one genome-wide significant association (HLA locus) compared to 22 in our UKB NFE GWAS from peripheral blood (**Fig. 3a**). Considering that these 22 associations are enriched at regulatory and coding regions of genes critical for immune regulation, we suggest that these additional analyses help reinforce that the EBV detected in peripheral blood is distinct from the reservoir identified in saliva.

**Methods**

*EBV DNAemia GWAS using saliva WGS samples*

Of the 33,168 individuals of EUR ancestry, 32,745 had full covariate information available (age < 100, sex_at_birth labeled as Male/Female, and genetic ancestry PCs 1-15). As we had no direct comparison for EBV viral load in the same tissue, we conducted the GWAS with three different models (**Supplementary Fig. 2g**):

- A binary trait of EBV DNA detection >1.2 EBV genomes per 10^4^ cells, which was the same threshold used in the blood analyses. We note that no serology data were available for the saliva cohort, making a data-driven threshold infeasible. Notably, as EBV DNA was far more abundant in saliva samples, ~50% of individuals were assigned as cases using this threshold.
- Next, we defined a binary trait that discriminated the top 10% load detected in saliva EUR samples (cases) relative to the remainder of the samples (controls). This was inspired by the rate of EBV DNAemia in blood.
- Finally, we used the EBV DNA levels as a quantitative trait in a standard linear model.

For common variant associations, we used plink1.9[^16^](https://paperpile.com/c/FN0JXJ/pWRZV) and the following filters for chr1-22: plink1.9 --mac 20 --geno 0.1 --maf 0.005 --hwe 1e-8, which resulted in a total of 12,253,919 variants. We performed GWAS with the same covariates (age, age^2^, sex, and ancestry PCs 1-15) as described in the main manuscript and obtained summary statistics for 11,882,778 variants using REGENIE[^17^](https://paperpile.com/c/FN0JXJ/gQk8y) (**Supplementary Fig. 2f**). We observed a strong signal at the HLA locus on chromosome 6, but no other genome-wide significant variants elsewhere (*P* < 5×10^-8^). The strong signal only in chromosome 6 was also observed for the two other models (**Supplementary Fig. 2g**).

*EBV DNAemia HLA regression using saliva WGS samples*

To examine whether the associated HLA alleles were similar between EBV DNAemia in blood and saliva, we performed HLA regression on individuals with EBV DNAemia in saliva samples. T1K[^18^](https://paperpile.com/c/FN0JXJ/ByoIE) was used to call HLA haplotypes for all 33,168 AoU EUR individuals, and regression was conducted on 32,748 individuals (age, sex_at_birth labeled as Male/Female, and genetic ancestry PCs 1-15) with the following covariates: age + sex + age^2^ + age*sex + age^2^*sex + PCs 1-15. For each tested HLA allele, Z-statistic for saliva EBV DNAemia were plotted against the scores for blood EBV DNAemia, subsetting to *P* < 0.1 for blood HLA associations (**Supplementary Fig. 3h**).

**Supplemental Note 3.** **EBV DNAemia is a biomarker of complex traits.**

We examined phenotypic associations with EBV DNAemia status using International Classification of Diseases (ICD)-10 codes in UKB and matching ICD-10CM codes in AoU, which are inherently limited by differences in hospital records[^19^](https://paperpile.com/c/FN0JXJ/pReD). Nevertheless, these codes provide an opportunity for discovery of novel associations between our EBV DNAemia biomarker and disease outcomes. Of the 141 significantly associated ICD-10 codes in UKB with sufficient representation in AoU, 87 (62%) replicated in AoU. Common phenotypes that replicated between biobanks included RA (UKB: OR = 1.46; *P* = 3.9×10^-26^; AoU: OR = 1.92; *P* = 6.6×10^-20^), COPD (UKB: OR = 1.98; *P* = 1.6×10^-33^; AoU: OR = 2.03; *P* = 8.4×10^-24^), and lung neoplasms (UKB: OR = 1.50; *P* = 6.7×10^-9^; AoU: OR = 1.87; *P* = 0.0062), as well as less-established phenotypes of peripheral vascular disease (UKB: OR = 1.47; *P* = 2.5×10^-22^; AoU: OR = 1.39; *P* = 3.1×10^-7^), emphysema (UKB: OR = 1.69; *P* = 1.1×10^-34^; AoU: OR = 1.65; *P* = 1.1×10^-9^), and tachycardia (UKB: OR = 1.30; *P* = 2.2×10^-9^; AoU: OR = 1.12; *P* = 0.029). We note that these phenotypic associations are correlative, and further work is needed to characterize the underlying causes of elevated persistent EBV DNA in the blood. The full list of tested UKB phenotypes and the resulting associations, as well as replicated results in AoU, can be found in **Supplementary Table 3**.

**Supplemental Note 4.** **Supporting analyses for UKB NFE genetic associations.**

From SNP-based heritability analyses of the UKB NFE GWAS summary statistics, we found heritability to be 2.21% (± 0.85%). For context, this SNP-based heritability estimate is similar in magnitude to prior reports of susceptibility to a broad class of viruses in population health registries[^20^](https://paperpile.com/c/FN0JXJ/YCqz5), as well as the heritability of severe COVID-19 following infection from SARS-CoV-2[^21^](https://paperpile.com/c/FN0JXJ/4AmWi).

As common in any immune-mediated GWAS, the strongest associations emerged near the MHC locus on chromosome 6[^22^](https://paperpile.com/c/FN0JXJ/UEyi). While the strong association in this region emphasizes the role of heterogeneous antigen processing and presentation in controlling EBV infection and viral persistence, direct mechanistic interpretations are difficult to derive from association results in this region[^23^](https://paperpile.com/c/FN0JXJ/nHRLj). Specifically, as the MHC locus is a hyper-polymorphic region with strong linkage disequilibrium, ascertaining causal variants at this locus is particularly challenging[^23^](https://paperpile.com/c/FN0JXJ/nHRLj). Annotation of ExWAS-associated variants with AlphaMissense[^24^](https://paperpile.com/c/FN0JXJ/wLoTz) helped refine candidate causal variants on chromosome 6 outside of HLA (**Supplementary Table 4**). Plausible functional variants included a missense mutation in *HFE* (rs1800562; C282Y; OR = 1.1; *P* = 2.16×10^-13^; **Supplementary Fig. 3a,b**) linked to iron storage disorders, consistent with prior reports of EBV modulating iron metabolism[^25^](https://paperpile.com/c/FN0JXJ/tptIo).

The strongest non-chromosome 6 association was in the region on chromosome 5 that encodes the aminopeptidases ERAP1, ERAP2, and LNPEP contiguously[^26,27^](https://paperpile.com/c/FN0JXJ/3G9Qe+GY0GQ), proteins that regulate effector immune responses following EBV infection[^28–30^](https://paperpile.com/c/FN0JXJ/Uake4+YdjPL+24wwq) (**Supplementary** **Fig. 3c**). Strongly associated variants in this region included rs2549794 (OR = 0.89, *P* = 3.61×10^-51^), an intronic variant in *ERAP2* that modulates gene expression[^31^](https://paperpile.com/c/FN0JXJ/afVwT) and manifests with pleiotropic effects on infectious respiratory disease and autoimmunity[^31^](https://paperpile.com/c/FN0JXJ/afVwT), consistent with the multi-faceted associations from our phenotypic associations (**Fig. 2**). Additionally, a strong coding variant (rs2476601; R620W) in *PTPN22*, a key immune regulator involved in T cell receptor signaling and type I interferon production[^32^](https://paperpile.com/c/FN0JXJ/rzl5U), was associated with EBV DNAemia (OR = 1.08, *P* = 1.07×10^-9^) (**Supplementary Fig. 3d**). Notably, the *PTPN22* R620W variant has been extensively characterized in autoimmune diseases, including RA, SLE, and type 1 diabetes[^33^](https://paperpile.com/c/FN0JXJ/IxqQK). In addition, this variant has been associated with susceptibility and severity of infectious diseases, including bacterial and viral infections[^34^](https://paperpile.com/c/FN0JXJ/pft4N). Moreover, missense variation in *SH2B3* (rs3184504; R262W; OR = 0.96, *P* = 2.43×10^-9^; **Supplementary Fig. 3e**) has been associated with Celiac disease and linked to type 1 diabetes, peripheral arterial disease, and coronary artery disease, as well as susceptibility to MS[^35–39^](https://paperpile.com/c/FN0JXJ/e6xGT+D5vr8+LPish+V3wSU+NCTtL). Functional studies of the R262W variant suggest that *SH2B3* mediates repression of IL12 signaling, promoting enhanced IFN-γ production and hypertension-associated pathology[^40^](https://paperpile.com/c/FN0JXJ/IUCDk). Other significant non-coding associations with rheumatoid arthritis included rs6679677, located between *RSBN1* and *PHTF1* and 52kb downstream of PTPN22 (OR = 0.93, *P* = 7.41×10^-10^), as well as rs3806624 (OR = 0.95, *P* = 6.67×10^-11^) and rs9880772 (OR = 0.95, *P* = 1.82×10^-11^), positioned in an intergenic region near *LINC01980* and *EOMES*, a gene that regulates antiviral CD4 T cell responses[^41^](https://paperpile.com/c/FN0JXJ/fvC2Q). Mechanistically, the non-coding variant rs3806624 is an expression Quantitative Trait Locus (eQTL) for *EOMES* from GTEx with risk alleles leading to decreased *EOMES* expression[^42^](https://paperpile.com/c/FN0JXJ/bmNfa). AlphaGenome[^43^](https://paperpile.com/c/FN0JXJ/niQDq) prediction of variant effect on chromatin also supports the role of rs3806624 as a repressive variant flanking TSS upstream of *EOMES* (**Supplementary Fig. 3f**). Furthermore, rs3806624 and rs9880772 have also been linked to lymphoid malignancies, including Hodgkin’s lymphoma[^44^](https://paperpile.com/c/FN0JXJ/nUug4) and chronic lymphocytic leukemia[^45^](https://paperpile.com/c/FN0JXJ/5p7tT), respectively.

While these anecdotes support the biological utility of our study, the full set of significantly associated loci is in **Supplementary Table 4**, and interactive visualization of the UKB NFE GWAS summary statistics can be found at <https://my.locuszoom.org/gwas/409414/?token=6385c90400414f34b8ed17679bf1495b>. We note that following an earlier version of our manuscript as a preprint, complementary work from two other groups reported analyses that supported our reports of a polygenic basis to EBV DNA persistence[^46,47^](https://paperpile.com/c/FN0JXJ/NSQI+Qm22). Collectively, our analyses reinforce that identifying and quantifying viral DNA in population-scale sequencing can yield direct insights into the genetic architecture and phenotypic correlations of viral DNA persistence at a population scale.

**Supplemental Note 5. Meta-analysis of UKB EBV DNAemia GWAS with diverse ancestries.**

As representation from diverse genetic ancestries enables greater power to detect additional genetic associations[^48^](https://paperpile.com/c/FN0JXJ/zWOTm), we performed a meta-analysis that evaluated the NFE results together with the five other genetic ancestries present in UKB, including individuals of Ashkenazi Jewish (ASJ; *n* = 2,636), East Asian (EAS; *n* = 2,108), South Asian (SAS; *n* = 9,168), Hispanic or Latin American (AMR: *n* = 619), and African (AFR; *n* = 8,147) ancestries. We identified 3 loci that were genome-wide significant in the meta-analysis but not in the NFE-only GWAS, including variants near *BIM*[*^49^*](https://paperpile.com/c/FN0JXJ/hczje) and *GSDMB*[*^50^*](https://paperpile.com/c/FN0JXJ/9KaLY) (**Supplementary Fig. 4a**). These genes regulate apoptosis during both innate and adaptive immune system development and function. We also observed variants overlapping the *TERT* locus, supporting evidence for an interaction between EBV and telomere regulation to preserve latency[^51^](https://paperpile.com/c/FN0JXJ/4p00K). Motivated by this increased statistical power, we further expanded the meta-analysis to include three additional genetic ancestries from AoU (EUR: *n =* 131,938; AFR: *n* = 56,911; AMR: *n* = 45,034), spanning 685,085 individuals and 9 total populations, which yielded an additional 20 novel genome-wide significant loci (**Supplementary Fig. 4b**). These loci included associations near immune-associated transcription factors (*BCL11A, MYC, ETS1*) and surface receptors (*CD160, KLRC1, TRAF3)*, underscoring the increase in statistical power by combining EBV DNAemia GWAS across heterogeneous populations and geographical origins. These results reinforce the value of profiling individuals of diverse ancestries, both for resolving other infectious agents as well as genetic variation that can be uniquely associated from non-European populations. More generally, our EBV analyses suggest that this platform for studying genetic determinants of infectious disease may be applicable across the human virome[^52^](https://paperpile.com/c/FN0JXJ/90CKp), and in turn, may further resolve the etiology of other complex traits.
 As a contrast to our genetic associations of EBV DNAemia, we conducted a GWAS on binarized EBV serostatus from UKB NFE individuals[^9^](https://paperpile.com/c/FN0JXJ/vlc7a). While quantitative serology titers yielded a genome-wide significant association at the MHC locus[^9^](https://paperpile.com/c/FN0JXJ/vlc7a), our analysis of binarized EBV serostatus showed no genome-wide significant associations (**Supplementary Fig. 4c**). This result reinforces the utility of the EBV DNAemia phenotype to drive discovery of genetic determinants of persistent EBV DNA.

**Methods**

*Multi-ancestral GWAS in UKB*

For multi-ancestral GWAS analysis, we applied the same REGENIE workflow as was done for the UKB NFE ancestry group to generate GWAS summary statistics for SAS, AFR, EAS, ASJ, and AMR cohorts in UKB. We used METAL[^53^](https://paperpile.com/c/FN0JXJ/qJESU) to perform inverse variant weighting meta-analyses across all ancestries. We then selected significant variants (*P*_meta_ < 5×10^−8^), removing those that were present in a single broad genetic ancestry, using these to define loci and index variants as described earlier and assessing these for overlap with NFE loci. Lastly, we executed a second meta-analysis combining the five UKB genetic ancestry groups with the three most abundant from AoU, including individuals of AFR (*n* = 56,911), AMR (*n* = 45,034), and EUR (*n* = 131,938) ancestry.

**Supplemental Note 6. Genetic diversity in EBV genomes across UKB and AoU.**

*Genetic diversity in EBV genomes across UKB and AoU*

Alongside host genetic variation, our framework enables genome-to-genome analyses[^54–56^](https://paperpile.com/c/FN0JXJ/8srDO+ZKaEh+IBokm) whereby variation in the viral genome can be similarly quantified. This setting yields a composition across these biobanks that approximates the circulating genetic variation of EBV in the UK and the US. We developed a heuristic to estimate the ratio of type 1 to type 2 EBV, confirming that type 1 was the predominant strain on both continents[^57^](https://paperpile.com/c/FN0JXJ/WAMxP) (UKB: 94.8%; AoU: 89.3%; **Extended Data Fig. 7a; Methods**) and highlighting the high-quality nature of our data at nucleotide resolution. The overall allele frequencies between the two cohorts were highly correlated (*r* = 0.92), though we observed that UKB detected more EBV variants (*n* = 13,568) than AoU (*n* = 5,471), which could be attributed to the difference in sample sizes or may reflect differences in strain heterogeneity in these two countries (**Extended Data Fig. 7b**).

We also assessed the observed frequency of 31 previously reported EBV protein-altering mutations derived from patients with NPC. All but four variants were detected in one or both cohorts at an allele frequency of 10%. The exceptions were *BALF2* I613V (UKB: 1.7%; AoU: 3.3%), *RPMS* D51N (UKB: 0.19%; AoU: 0.69%), *BNRF1* P694H (UKB: 0.20%; AoU: 0.0%), and *BALF2* V317M (UKB: 0.18%; AoU: 0.0%). *BALF2* (I613V and V317M), a gene that is an essential component of the lytic cycle and acts as a DNA-binding protein that facilitates EBV DNA replication during reactivation. These two *BALF2* variants have been linked to high-risk NPC populations across multiple studies[^58^](https://paperpile.com/c/FN0JXJ/EbXJy). RPMS is a nuclear protein that associates with a component of the Notch pathway to repress Notch activity[^59^](https://paperpile.com/c/FN0JXJ/1RKQt), and the identified variant (D51N) is reported to result in a longer half-life of the protein[^60^](https://paperpile.com/c/FN0JXJ/cLlIj). Finally, BNRF1 is a tegument protein that prevents repressive histone loading onto EBV genomes and promotes efficient viral replication[^61^](https://paperpile.com/c/FN0JXJ/bbfX3). The P694H variant, to the best of our knowledge, has not been described with a functional role. We emphasize that our analyses do not necessarily validate the potential oncogenic role of these mutations. Instead, we highlight our approach to resolve population-level viral strain information that can be useful to mitigate geographical biases in interpreting variants of unknown significance in the EBV genome.

**SUPPLEMENTAL FIGURES**


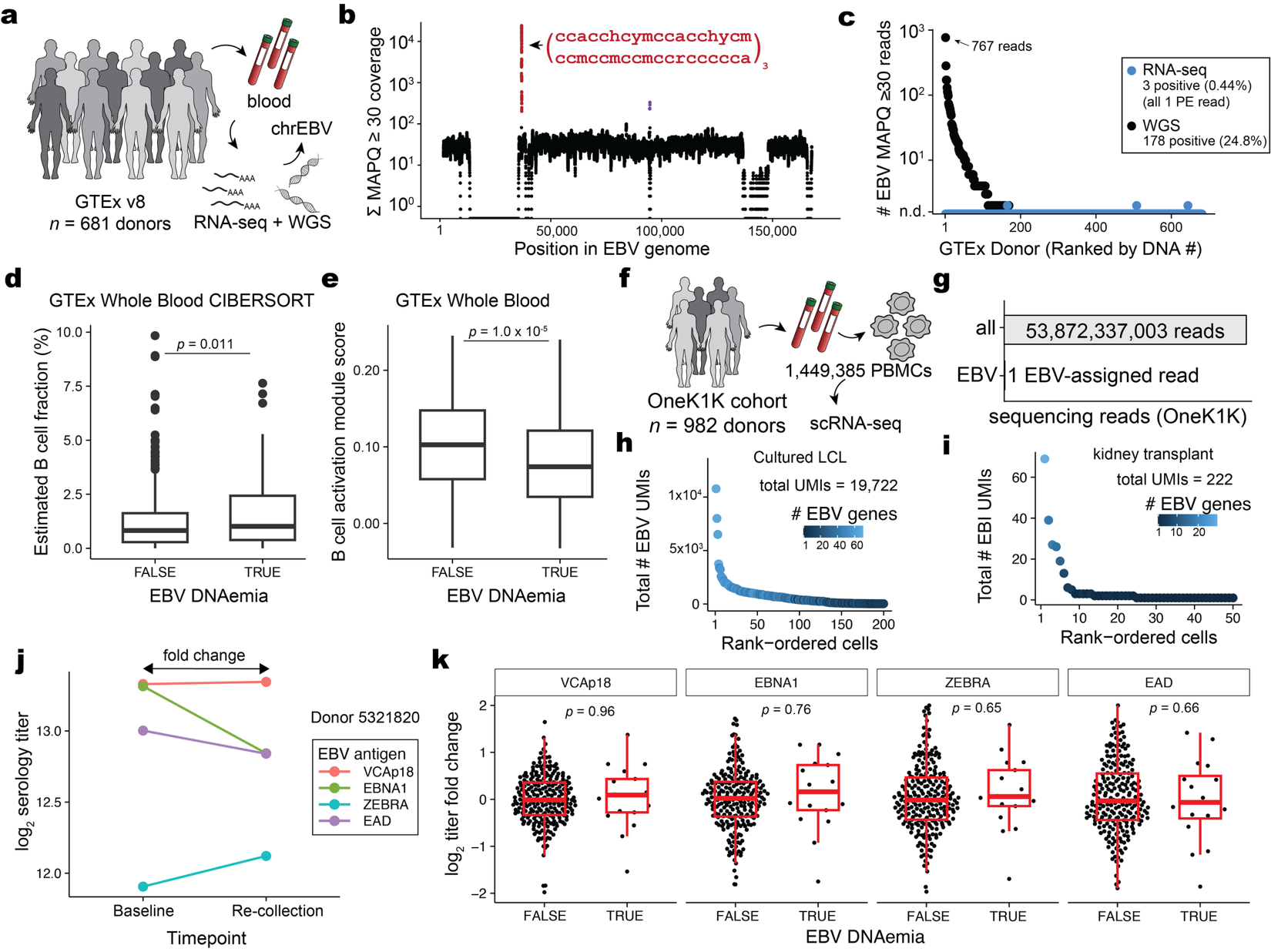


**Supplementary Fig. 1. Characterization of EBV latency in large genomics datasets. (a)** Schematic for the GTEx consortium, highlighting donors where matching WGS and RNA-seq data were available from peripheral blood. **(b)** Sum of per-base read coverage of high-confidence EBV-mapping reads. Two repetitive regions with inflated coverage are noted in red and purple. **(c)** Characterization of EBV detection from 681 GTEx donors with paired WGS and RNA-seq from peripheral blood. Three RNA-seq samples were positive, all with one paired-end (PE) read. **(d)** Estimated B cell fraction using RNA-seq deconvolution and cell type abundance estimation. Statistical test: Mann Whitney U Tests with two-sided hypothesis testing for *n* = 681 GTEx donors. **(e)**  B cell activation (Panther Pathway) module score per donor. Statistical test: Mann Whitney U Tests with two-sided hypothesis testing for *n* = 681 GTEx donors. **(f)** Schematic of data generation from the OneK1K cohort. **(g)** Summary of EBV quantification from the OneK1K cohort, showing total read number from the consortium (top) and number of EBV-assigned UMIs. **(h)** Summary of scRNA-seq results from a lymphoblastic cell line (LCL), a positive control for EBV transformation (SAMN34277123). **(i)** Summary of scRNA-seq results from a kidney transplant, a positive control for EBV reactivation (SAMN35232564). **(j)** Depiction of longitudinal serology measures of four EBV antigens for an exemplar EBV DNAemia donor. **(k)** Characterization of EBV longitudinal antigen titers across UKB, stratified by EBV DNAemia. Statistical test: two-sided Student’s *t-*test. Sample size: *n* = 262. Boxplots: center line, median; box limits, first and third quartiles; whiskers, 1.5× interquartile range.


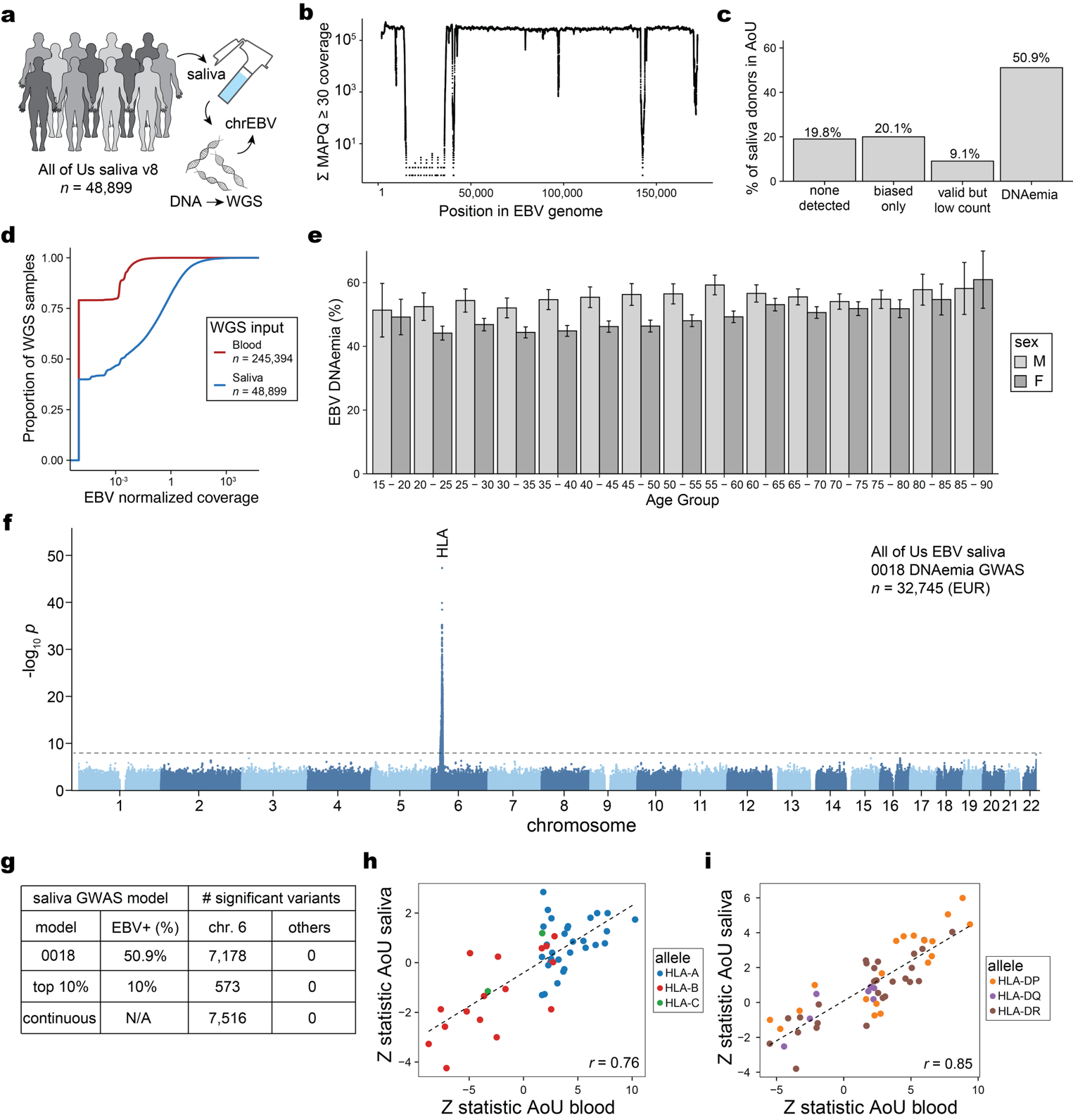


**Supplementary Fig. 2. Quantification of EBV DNA from AoU saliva WGS samples.** **(a)** Schematic of the approach. **(b)** Sum of per-base read coverage of high-confidence EBV-mapping reads. **(c)** Partition of AoU saliva WGS participants by EBV DNA detection after accounting for biased regions. “Biased only” refers to participants with reads mapping to only the two repetitive regions indicated in main text **Fig. 1b**. “Valid and low count” refers to participants with EBV DNA detected after masking the two biased regions. “DNAemia” refers to participants who pass the threshold of 1.2 EBV copies per 10^4^ human cells. **(d)** Empirical cumulative distribution of detected EBV DNA across all saliva samples, compared to blood. **(e)** Percent saliva EBV DNAemia resolved by sex and age in AoU. Statistical test: two-sided proportion test comparing sex in the associated age bin. Error bars: standard error of the mean. **(f)** Manhattan plot summarizing the genome-wide association statistics for saliva EBV DNAemia for 32,745 individuals of EUR ancestry. The only genome-wide significant association region in the HLA locus (*P* < 5×10⁻⁸) is annotated. **(g)** Number of genome-wide significant variants with each GWAS model, in chromosome 6 or not. “0018” refers to the threshold of 1.2 EBV copies per 10^4^ human cells. “Top 10%” refers to a threshold of ~1,800 copies per 10^4^ cells. “Continuous” refers to providing the EBV DNA load as a quantitative trait. **(h)** Results of allele-level regression, showing the z-statistic of the Wald test for individual HLA class I alleles, subsetted to *P* < 0.1 in AoU blood regression results. **(i)** Same as (h) but for class II alleles.

**
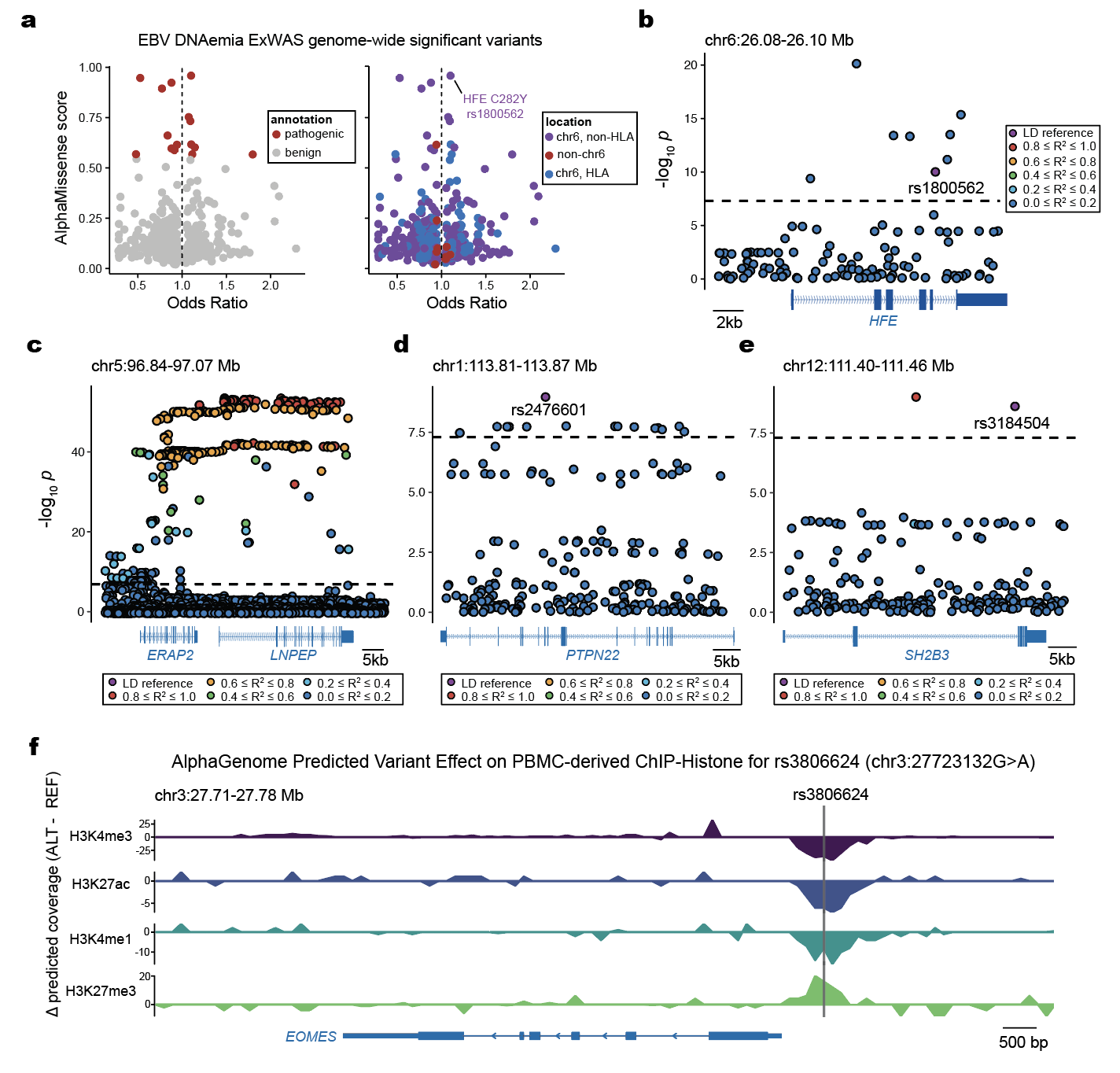

Supplementary Fig. 3. Supporting analyses for NFE genetic association studies. (a)** AlphaMissense characterization of significant missense variants implicated in the ExWAS association analyses. Left: AlphaMissense score and annotation; right: genomic positions of ExWAS variants. **(b)** Zoom plot of the *HFE* locus in the EBV DNAemia GWAS with the UKB NFE cohort, highlighting the rs1800562 variant. **(c)** Zoom plot of the *ERAP2-LNPEP* locus in the EBV DNAemia GWAS with the UKB NFE cohort. **(d)** Zoom plot of the *PTPN22* locus in the EBV DNAemia GWAS with the UKB NFE cohort, highlighting the rs2476601 variant. **(e)** Zoom plot of the *SH2B3* locus in the EBV DNAemia GWAS with the UKB NFE cohort, highlighting the rs3184504 variant. **(f)** AlphaGenome[^43^](https://paperpile.com/c/FN0JXJ/niQDq) predicted chromatin changes for the rs3806624 variant near the *EOMES* promoter. All predicted ChIP-seq effects are scored from pan-PBMC data. For all plots: *P* values are from a likelihood ratio test from logistic regression model (two-sided).

**
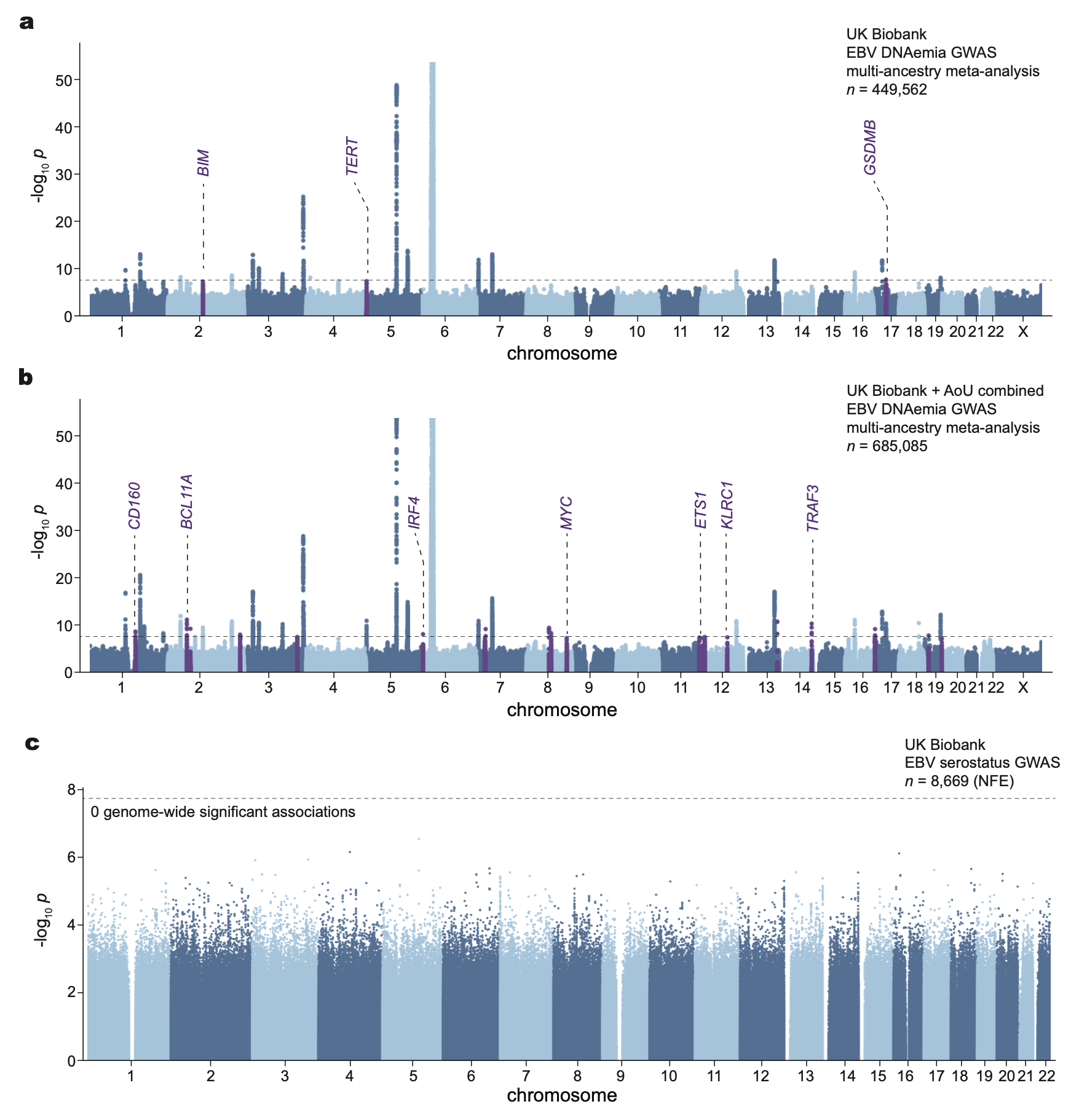
**

**Supplementary Fig. 4. Additional genetic association studies. (a)** Meta-analyses of 6 ancestries from UKB. Purple marks loci that were genome-wide significant (*P* < 5×10^-8^) here, but not in the NFE-only GWAS. **(b)** Meta-analysis of 6 genetic ancestries from UKB and an additional 3 genetic ancestries from AoU. Purple reflects loci that were genome-wide significant (*P* < 5×10^-8^) here, but not in the UKB meta-analysis GWAS or the NFE-only GWAS. **(c)** Manhattan plot of binarized EBV serostatus among 8,669 individuals of NFE ancestry in UKB. Statistical test: likelihood ratio test from logistic regression model (two-sided).

**REFERENCES**

1. [Damania, B., Kenney, S. C. & Raab-Traub, N. Epstein-Barr virus: Biology and clinical disease. *Cell* **185**, 3652–3670 (2022).](http://paperpile.com/b/FN0JXJ/dITGP)

2. [Yazar, S. *et al.* Single-cell eQTL mapping identifies cell type-specific genetic control of autoimmune disease. *Science* **376**, eabf3041 (2022).](http://paperpile.com/b/FN0JXJ/9eGFz)

3. [Bristol, J. A. *et al.* Reduced IRF4 expression promotes lytic phenotype in Type 2 EBV-infected B cells. *PLoS Pathog.* **18**, e1010453 (2022).](http://paperpile.com/b/FN0JXJ/AhPS1)

4. [Shi, T. *et al.* Single-cell transcriptomic analysis of renal allograft rejection reveals insights into intragraft TCR clonality. *J. Clin. Invest.* **133**, (2023).](http://paperpile.com/b/FN0JXJ/Ifq5h)

5. [Lareau, C. A. *et al.* Latent human herpesvirus 6 is reactivated in CAR T cells. *Nature* **623**, 608–615 (2023).](http://paperpile.com/b/FN0JXJ/Rw7q3)

6. [Babcock, G. J., Hochberg, D. & Thorley-Lawson, A. D. The expression pattern of Epstein-Barr virus latent genes in vivo is dependent upon the differentiation stage of the infected B cell. *Immunity* **13**, 497–506 (2000).](http://paperpile.com/b/FN0JXJ/u40N2)

7. [Babcock, G. J., Decker, L. L., Freeman, R. B. & Thorley-Lawson, D. A. Epstein-barr virus-infected resting memory B cells, not proliferating lymphoblasts, accumulate in the peripheral blood of immunosuppressed patients. *J. Exp. Med.* **190**, 567–576 (1999).](http://paperpile.com/b/FN0JXJ/CFhk9)

8. [Younis, S. *et al.* Epstein-Barr virus reprograms autoreactive B cells as antigen-presenting cells in systemic lupus erythematosus. *Sci. Transl. Med.* **17**, eady0210 (2025).](http://paperpile.com/b/FN0JXJ/alQr)

9. [Mentzer, A. J. *et al.* Identification of host–pathogen-disease relationships using a scalable multiplex serology platform in UK Biobank. *Nature Communications* **13**, 1–12 (2022).](http://paperpile.com/b/FN0JXJ/vlc7a)

10. [Chen, B., Khodadoust, M. S., Liu, C. L., Newman, A. M. & Alizadeh, A. A. Profiling tumor infiltrating immune cells with CIBERSORT. *Methods Mol. Biol.* **1711**, 243–259 (2018).](http://paperpile.com/b/FN0JXJ/WE0yZ)

11. [Hao, Y. *et al.* Integrated analysis of multimodal single-cell data. *Cell* **184**, 3573–3587.e29 (2021).](http://paperpile.com/b/FN0JXJ/eJYIa)

12. [Sullivan, D. K. *et al.* kallisto, bustools and kb-python for quantifying bulk, single-cell and single-nucleus RNA-seq. *Nat. Protoc.* **20**, 587–607 (2025).](http://paperpile.com/b/FN0JXJ/NEjIG)

13. [Brenner, N. *et al.* Validation of Multiplex Serology detecting human herpesviruses 1-5. *PLoS One* **13**, e0209379 (2018).](http://paperpile.com/b/FN0JXJ/SS3pU)

14. [Boldogh, I., Albrecht, T. & Porter, D. D. Persistent viral infections. in *Medical Microbiology* (University of Texas Medical Branch at Galveston, Galveston (TX), 1996).](http://paperpile.com/b/FN0JXJ/3QqAy)

15. [*1000G Project EBV Processing*. (https://github.com/clareaulab/ebv_biobank_gwas/blob/main/mwe-1000G/01_1000G_EBV.ipynb).](http://paperpile.com/b/FN0JXJ/g1iml)

16. [Purcell, S. *et al.* PLINK: a tool set for whole-genome association and population-based linkage analyses. *Am. J. Hum. Genet.* **81**, 559–575 (2007).](http://paperpile.com/b/FN0JXJ/pWRZV)

17. [Mbatchou, J. *et al.* Computationally efficient whole-genome regression for quantitative and binary traits. *Nat. Genet.* **53**, 1097–1103 (2021).](http://paperpile.com/b/FN0JXJ/gQk8y)

18. [Song, L., Bai, G., Liu, X. S., Li, B. & Li, H. Efficient and accurate KIR and HLA genotyping with massively parallel sequencing data. *Genome Res.* **33**, 923–931 (2023).](http://paperpile.com/b/FN0JXJ/ByoIE)

19. [Levine, K. S. *et al.* Virus exposure and neurodegenerative disease risk across national biobanks. *Neuron* **111**, 1086–1093.e2 (2023).](http://paperpile.com/b/FN0JXJ/pReD)

20. [Nudel, R. *et al.* A large-scale genomic investigation of susceptibility to infection and its association with mental disorders in the Danish population. *Transl. Psychiatry* **9**, 283 (2019).](http://paperpile.com/b/FN0JXJ/YCqz5)

21. [COVID-19 Host Genetics Initiative. A second update on mapping the human genetic architecture of COVID-19. *Nature* **621**, E7–E26 (2023).](http://paperpile.com/b/FN0JXJ/4AmWi)

22. [Kennedy, A. E., Ozbek, U. & Dorak, M. T. What has GWAS done for HLA and disease associations? *Int. J. Immunogenet.* **44**, 195–211 (2017).](http://paperpile.com/b/FN0JXJ/UEyi)

23. [Kulski, J. K., Suzuki, S. & Shiina, T. Human leukocyte antigen super-locus: nexus of genomic supergenes, SNPs, indels, transcripts, and haplotypes. *Hum. Genome Var.* **9**, 49 (2022).](http://paperpile.com/b/FN0JXJ/nHRLj)

24. [Cheng, J. *et al.* Accurate proteome-wide missense variant effect prediction with AlphaMissense. *Science* **381**, eadg7492 (2023).](http://paperpile.com/b/FN0JXJ/wLoTz)

25. [Zhao, X. *et al.* Epstein-Barr virus modulates iron metabolism and ferritin expression to promote tumorigenesis in gastric cancer. *J. Mol. Histol.* **56**, 231 (2025).](http://paperpile.com/b/FN0JXJ/tptIo)

26. [Paladini, F., Fiorillo, M. T., Tedeschi, V., Mattorre, B. & Sorrentino, R. The multifaceted nature of aminopeptidases ERAP1, ERAP2, and LNPEP: From evolution to disease. *Front. Immunol.* **11**, 1576 (2020).](http://paperpile.com/b/FN0JXJ/3G9Qe)

27. [Evans, D. M. *et al.* Interaction between ERAP1 and HLA-B27 in ankylosing spondylitis implicates peptide handling in the mechanism for HLA-B27 in disease susceptibility. *Nature Genetics* **43**, 761–767 (2011).](http://paperpile.com/b/FN0JXJ/GY0GQ)

28. [Tsao, H.-W. *et al.* Targeting the aminopeptidase ERAP enhances antitumor immunity by disrupting the NKG2A-HLA-E inhibitory checkpoint. *Immunity* **57**, 2863–2878.e12 (2024).](http://paperpile.com/b/FN0JXJ/Uake4)

29. [Vietzen, H. *et al.* HLA-E-restricted immune responses are crucial for the control of EBV infections and the prevention of PTLD. *Blood* **141**, 1560–1573 (2023).](http://paperpile.com/b/FN0JXJ/YdjPL)

30. [Vietzen, H. *et al.* Ineffective control of Epstein-Barr-virus-induced autoimmunity increases the risk for multiple sclerosis. *Cell* **186**, 5705–5718.e13 (2023).](http://paperpile.com/b/FN0JXJ/24wwq)

31. [Hamilton, F. *et al.* Variation in ERAP2 has opposing effects on severe respiratory infection and autoimmune disease. *Am. J. Hum. Genet.* **110**, 691–702 (2023).](http://paperpile.com/b/FN0JXJ/afVwT)

32. [Wang, Y. *et al.* The autoimmunity-associated gene PTPN22 potentiates toll-like receptor-driven, type 1 interferon-dependent immunity. *Immunity* **39**, 111–122 (2013).](http://paperpile.com/b/FN0JXJ/rzl5U)

33. [Stanford, S. M. & Bottini, N. PTPN22: the archetypal non-HLA autoimmunity gene. *Nat. Rev. Rheumatol.* **10**, 602–611 (2014).](http://paperpile.com/b/FN0JXJ/IxqQK)

34. [Bottini, N. & Peterson, E. J. Tyrosine phosphatase PTPN22: multifunctional regulator of immune signaling, development, and disease. *Annu. Rev. Immunol.* **32**, 83–119 (2014).](http://paperpile.com/b/FN0JXJ/pft4N)

35. [Hunt, K. A. *et al.* Newly identified genetic risk variants for celiac disease related to the immune response. *Nat. Genet.* **40**, 395–402 (2008).](http://paperpile.com/b/FN0JXJ/e6xGT)

36. [Lian, J. *et al.* Meta-analyses of four eosinophil related gene variants in coronary heart disease. *J. Thromb. Thrombolysis* **36**, 394–401 (2013).](http://paperpile.com/b/FN0JXJ/D5vr8)

37. [Kullo, I. J. *et al.* The ATXN2-SH2B3 locus is associated with peripheral arterial disease: an electronic medical record-based genome-wide association study. *Front. Genet.* **5**, 166 (2014).](http://paperpile.com/b/FN0JXJ/LPish)

38. [Todd, J. A. *et al.* Robust associations of four new chromosome regions from genome-wide analyses of type 1 diabetes. *Nat. Genet.* **39**, 857–864 (2007).](http://paperpile.com/b/FN0JXJ/V3wSU)

39. [Alcina, A. *et al.* The autoimmune disease-associated KIF5A, CD226 and SH2B3 gene variants confer susceptibility for multiple sclerosis. *Genes Immun.* **11**, 439–445 (2010).](http://paperpile.com/b/FN0JXJ/NCTtL)

40. [Alexander, M. R. *et al.* A single nucleotide polymorphism in SH2B3/LNK promotes hypertension development and renal damage. *Circ. Res.* **131**, 731–747 (2022).](http://paperpile.com/b/FN0JXJ/IUCDk)

41. [Dhume, K., Kaye, B. & McKinstry, K. K. Regulation of CD4 T cell responses by the transcription factor eomesodermin. *Biomolecules* **12**, 1549 (2022).](http://paperpile.com/b/FN0JXJ/fvC2Q)

42. [GTEx Consortium. The GTEx Consortium atlas of genetic regulatory effects across human tissues. *Science* **369**, 1318–1330 (2020).](http://paperpile.com/b/FN0JXJ/bmNfa)

43. [Avsec, Ž. *et al.* AlphaGenome: advancing regulatory variant effect prediction with a unified DNA sequence model. *Bioinformatics* (2025).](http://paperpile.com/b/FN0JXJ/niQDq)

44. [Frampton, M. *et al.* Variation at 3p24.1 and 6q23.3 influences the risk of Hodgkin’s lymphoma. *Nat. Commun.* **4**, 2549 (2013).](http://paperpile.com/b/FN0JXJ/nUug4)

45. [Berndt, S. I. *et al.* Meta-analysis of genome-wide association studies discovers multiple loci for chronic lymphocytic leukemia. *Nat. Commun.* **7**, 10933 (2016).](http://paperpile.com/b/FN0JXJ/5p7tT)

46. [Schmidt, A. *et al.* Host control of latent Epstein-Barr virus infection. *Infectious Diseases (except HIV/AIDS)* (2025).](http://paperpile.com/b/FN0JXJ/NSQI)

47. [Kamitaki, N., Tang, D., McCarroll, S. A. & Loh, P.-R. Genes and environment profoundly affect the human virome. *bioRxivorg* 2025.09.08.674901 (2025) doi:](http://paperpile.com/b/FN0JXJ/Qm22)[10.1101/2025.09.08.674901](http://dx.doi.org/10.1101/2025.09.08.674901)[.](http://paperpile.com/b/FN0JXJ/Qm22)

48. [Peterson, R. E. *et al.* Genome-wide association studies in ancestrally diverse populations: Opportunities, methods, pitfalls, and recommendations. *Cell* **179**, 589–603 (2019).](http://paperpile.com/b/FN0JXJ/zWOTm)

49. [Akiyama, T. & Tanaka, S. Bim: guardian of tissue homeostasis and critical regulator of the immune system, tumorigenesis and bone biology. *Arch. Immunol. Ther. Exp. (Warsz.)* **59**, 277–287 (2011).](http://paperpile.com/b/FN0JXJ/hczje)

50. [Hu, Y., Jin, S., Cheng, L., Liu, G. & Jiang, Q. Autoimmune disease variants regulate GSDMB gene expression in human immune cells and whole blood. *Proc. Natl. Acad. Sci. U. S. A.* **114**, E7860–E7862 (2017).](http://paperpile.com/b/FN0JXJ/9KaLY)

51. [Giunco, S. *et al.* Cross talk between EBV and telomerase: the role of TERT and NOTCH2 in the switch of latent/lytic cycle of the virus. *Cell Death Dis.* **6**, e1774 (2015).](http://paperpile.com/b/FN0JXJ/4p00K)

52. [Sasa, N. *et al.* Blood DNA virome associates with autoimmune diseases and COVID-19. *Nat. Genet.* **57**, 65–79 (2025).](http://paperpile.com/b/FN0JXJ/90CKp)

53. [Willer, C. J., Li, Y. & Abecasis, G. R. METAL: fast and efficient meta-analysis of genomewide association scans. *Bioinformatics* **26**, 2190–2191 (2010).](http://paperpile.com/b/FN0JXJ/qJESU)

54. [Ansari, M. A. *et al.* Genome-to-genome analysis highlights the effect of the human innate and adaptive immune systems on the hepatitis C virus. *Nat. Genet.* **49**, 666–673 (2017).](http://paperpile.com/b/FN0JXJ/8srDO)

55. [Johansen, J. *et al.* Genome binning of viral entities from bulk metagenomics data. *Nat. Commun.* **13**, 965 (2022).](http://paperpile.com/b/FN0JXJ/ZKaEh)

56. [Xu, M. *et al.* Host genetic variants, Epstein-Barr virus subtypes, and the risk of nasopharyngeal carcinoma: Assessment of interaction and mediation. *Cell Genom.* **4**, 100474 (2024).](http://paperpile.com/b/FN0JXJ/IBokm)

57. [Correia, S. *et al.* Natural variation of Epstein-Barr virus genes, proteins, and primary MicroRNA. *J. Virol.* **91**, (2017).](http://paperpile.com/b/FN0JXJ/WAMxP)

58. [Briercheck, E. L. *et al.* Geographic EBV variants confound disease-specific variant interpretation and predict variable immune therapy responses. *Blood advances* **8**, (2024).](http://paperpile.com/b/FN0JXJ/EbXJy)

59. [Zhang, J., Chen, H., Weinmaster, G. & Hayward, S. D. Epstein-Barr virus BamHi-a rightward transcript-encoded RPMS protein interacts with the CBF1-associated corepressor CIR to negatively regulate the activity of EBNA2 and NotchIC. *J. Virol.* **75**, 2946–2956 (2001).](http://paperpile.com/b/FN0JXJ/1RKQt)

60. [Feng, F.-T. *et al.* A single nucleotide polymorphism in the Epstein-Barr virus genome is strongly associated with a high risk of nasopharyngeal carcinoma. *Chin. J. Cancer* **34**, 563–572 (2015).](http://paperpile.com/b/FN0JXJ/cLlIj)

61. [Sagou, K. *et al.* Epstein-Barr virus lytic gene BNRF1 promotes B-cell lymphomagenesis via IFI27 upregulation. *PLoS Pathog.* **20**, e1011954 (2024).](http://paperpile.com/b/FN0JXJ/bbfX3)
