# Supplementary material for: Population-scale sequencing resolves determinants of persistent EBV DNA
Source: Nature. 2026 Jan 28;650(8102):664–72. doi: 10.1038/s41586-025-10020-2 (PMC12888827; doi:10.1038/s41586-025-10020-2)
Supplement: Supplementary file 2 — Reporting Summary [file 41586_2025_10020_MOESM2_ESM.pdf]

nature portfolio

Corresponding author(s): Petrovski, Dhindsa, Lareau

Last updated by author(s): Nov 19, 2025

Reporting Summary

Nature Portfolio wishes to improve the reproducibility of the work that we publish. This form provides structure for consistency and transparency in reporting. For further information on Nature Portfolio policies, see our Editorial Policies and the Editorial Policy Checklist.

Statistics

For all statistical analyses, confirm that the following items are present in the figure legend, table legend, main text, or Methods section.

n/a	Confirmed	
☐	☒	The exact sample size (*n*) for each experimental group/condition, given as a discrete number and unit of measurement
☐	☒	A statement on whether measurements were taken from distinct samples or whether the same sample was measured repeatedly
☐	☒	The statistical test(s) used AND whether they are one- or two-sided<br>*Only common tests should be described solely by name; describe more complex techniques in the Methods section.*
☐	☒	A description of all covariates tested
☐	☒	A description of any assumptions or corrections, such as tests of normality and adjustment for multiple comparisons
☐	☒	A full description of the statistical parameters including central tendency (e.g. means) or other basic estimates (e.g. regression coefficient) AND variation (e.g. standard deviation) or associated estimates of uncertainty (e.g. confidence intervals)
☐	☒	For null hypothesis testing, the test statistic (e.g. *F*, *t*, *r*) with confidence intervals, effect sizes, degrees of freedom and *P* value noted<br>*Give P values as exact values whenever suitable.*
☒	☐	For Bayesian analysis, information on the choice of priors and Markov chain Monte Carlo settings
☒	☐	For hierarchical and complex designs, identification of the appropriate level for tests and full reporting of outcomes
☐	☒	Estimates of effect sizes (e.g. Cohen's *d*, Pearson's *r*), indicating how they were calculated

Our web collection on statistics for biologists contains articles on many of the points above.

Software and code

Policy information about availability of computer code

Data collection	Reads mapping to the chrEBV contig were extracted for the UKB using samtools v1.17 and for the AoU cohort using GATK v4.2.6. Four-digit HLA calls were acquired from the UKB RAP web portal. HLA genotypes for AoU were inferred via T1K v1.0.7. For both cohorts, the underlying composition of the viral genomes were determined using bam-readcount v1.0.1 on the merged .bam file of all chrEBV reads.
Data analysis	Downstream analyses were performed using bowtie2 v2.5.1, REGENIE v3.2.4 (AoU) and v3.5 (UKB), plink v1.9 (AoU) and 2.0 (UKB), ldsc v1.0.1, GenomicRanges v1.59.0, AlphaMissense v2023.hg38, cupcake v0.1.0, CIBERSORT v1.0.6, kallisto v0.50.0, Seurat v5, clusterProfiler v4.0, chromVAR v1.5.0, NetMHCpan v4.1, and NetMHCIIpan v4.3. Code to reproduce custom analyses in this manuscript is available online at https://github.com/clareaulab/ebv_biobank_gwas.

For manuscripts utilizing custom algorithms or software that are central to the research but not yet described in published literature, software must be made available to editors and reviewers. We strongly encourage code deposition in a community repository (e.g. GitHub). See the Nature Portfolio guidelines for submitting code & software for further information.

Data

Policy information about availability of data

All manuscripts must include a data availability statement. This statement should provide the following information, where applicable:

- Accession codes, unique identifiers, or web links for publicly available datasets
- A description of any restrictions on data availability
- For clinical datasets or third party data, please ensure that the statement adheres to our policy

The UK Biobank data are available to qualified researchers (please refer to the details at http://www.ukbiobank.ac.uk/register-apply/). The All of Us data are available as a featured workspace to registered researchers of the All of Us Researcher Workbench (https://www.researchallofus.org/). Summary statistics from the EBV DNAemia discovery NFE GWAS in UKB are available at https://my.locuszoom.org/gwas/409414/?token=6385c90400414f34b8ed17679bf1495b and have been uploaded to the GWAS catalogue (GCST90572743). No new sequencing data was generated as part of this study.

Research involving human participants, their data, or biological material

Policy information about studies with human participants or human data. See also policy information about sex, gender (identity/presentation), and sexual orientation and race, ethnicity and racism.

Reporting on sex and gender	All analyses included males and females. We report that sex was used as a covariate in many downstream analyses and association tests.
Reporting on race, ethnicity, or other socially relevant groupings	Both sex assigned at birth and self reported gender of individuals was collected. Sex assigned at birth was used for all relevant analyses, including only individuals where the genetically inferred sex matched sex assigned at birth.
Population characteristics	For the discovery cohort, the average age was 57, and 54% of the cohort was female. 94% of the cohort is of European ancestry (UK Biobank). For the All of Us cohort, adults 18 years and older who have the capacity to consent and currently reside in the U.S. or a U.S. territory were eligible.
Recruitment	Participants were recruited to the UK Biobank on a voluntary basis. Approx 500K individuals 40-69 years of age in 2006-2010 volunteered. Informed consent was obtained for all participants. It has previously been observed that participants are less likely to live in socioeconomically deprived areas than non-participants, and they tend to be healthier than non-participants, which may impact some of the reporting rates in comparison to what could be observed through random sampling from the UK population. Fry et al (10.1093/aje/kwx246). Recruitment of the All of Us Research Program was described in detail in "The “All of Us” Research Program", NEJM 2019; briefly individuals were recruited through direct participant enrollment or recruitment at one of >340 locations at US healthcare provider organizations or federally qualified community health centers.
Ethics oversight	The protocols for UK Biobank are overseen by The UK Biobank Ethics Advisory Committee (EAC), for more information see https://www.ukbiobank.ac.uk/ethics/ and https://www.ukbiobank.ac.uk/wp-content/up1oads/2011/05/EGF20082.pdf Informed consent for the All of Us participants is conducted in person or through an eConsent platform that includes primary consent, HIPAA Authorization for Research EHRs, and Consent for Return of Genomic Results. The protocol was reviewed by the Institutional Review Board (IRB) of the All of Us Research Program. The All of Us IRB follows the regulations and guidance of the NIH Office for Human Research Protections for all studies, ensuring that the rights and welfare of research participants are overseen and protected uniformly.

Note that full information on the approval of the study protocol must also be provided in the manuscript.

Field-specific reporting

Please select the one below that is the best fit for your research. If you are not sure, read the appropriate sections before making your selection.

☒ Life sciences ☐ Behavioural & social sciences ☐ Ecological, evolutionary & environmental sciences

For a reference copy of the document with all sections, see nature.com/documents/nr-reporting-summary-flat.pdf

Life sciences study design

All studies must disclose on these points even when the disclosure is negative.

Sample size	No pre-determined sample size was calculated for these analyses as analyses were retrospective from large cohorts. The sample sizes for genetic and phenotypic associations exceeded 490,000 from the UKB (discovery cohort) and 245,000 from AoU (replication cohort) represent the largest cohorts to date to study the genetic basis of EBV (a minimum ~50x increase from any past study), meaning our sample size was substantially larger than any published analysis to date.
Data exclusions	No data or individuals with successful generation of genome sequencing data were excluded from these analyses.
Replication	The UK Biobank cohort was used for discovery. The All of Us cohort was used for replication studies. The GWAS and PheWAS results showed largely concordant results for variants and phecodes that could be analyzed in both cohorts. For PheWAS, 87 of 141 (62%) significant

	phecodes in UKB that could be remapped to the AoU phecodes replicated in AoU ($P < 0.05$; OR directionally concordant with UKB statistics). For GWAS, 40,675 variants were genome-wide significant ($P < 5\times10^{-8}$) in UKB and passed quality control filters in AoU, of which 91.4% were replicated in AoU (nominal $P < 0.05$; OR concordant).
Randomization	This study is observational. Randomization was not applicable to this study.
Blinding	This study is observational, using coded de-identified data. Blinding was not applicable to this study

Reporting for specific materials, systems and methods

We require information from authors about some types of materials, experimental systems and methods used in many studies. Here, indicate whether each material, system or method listed is relevant to your study. If you are not sure if a list item applies to your research, read the appropriate section before selecting a response.

Materials & experimental systems

n/a	Involved in the study
☒	☐ Antibodies
☒	☐ Eukaryotic cell lines
☒	☐ Palaeontology and archaeology
☒	☐ Animals and other organisms
☒	☐ Clinical data
☒	☐ Dual use research of concern
☒	☐ Plants

Methods

n/a	Involved in the study
☒	☐ ChIP-seq
☒	☐ Flow cytometry
☒	☐ MRI-based neuroimaging

Plants

Seed stocks	N/A
Novel plant genotypes	N/A
Authentication	N/A
