# Supplementary material for: Population-scale sequencing resolves determinants of persistent EBV DNA
Source: Nature. 2026 Jan 28;650(8102):664–72. doi: 10.1038/s41586-025-10020-2 (PMC12888827; doi:10.1038/s41586-025-10020-2)
Supplement: Supplementary file 3 — Supplementary Tables 1–8 [file 41586_2025_10020_MOESM3_ESM.zip › 2025-05-12670D-s3/Supplementary-Table-legends.docx]

**Supplementary Information Guide for**

# **Population-scale sequencing resolves determinants of persistent EBV DNA**

**SUPPLEMENTARY TABLES**

**Supplementary Table 1.** Crosstabs of EBV serostatus and DNAemia with various covariates in UKB.

**Supplementary Table 2.** Full list of immunosuppressive drug codes in UKB and AoU.

**Supplementary Table 3.** PheWAS summary statistics: **(a)** Full list of binary and quantitative traits tested in UKB; **(b)** Summary of UKB PheWAS; **(c)** Reproducible PheWAS associations between UKB and AoU.

**Supplementary Table 4.** Summary of significant loci and relevant annotations from genetic association studies. **(a)** Summary of UKB NFE GWAS with ExWAS and AoU validation annotations; **(b)** Summary of all ExWAS associations from UKB NFE, including AlphaMissense scores; **(c)** Additional loci discovered via UKB multi-ancestry meta-analysis; **(d)** Additional loci discovered via UKB+AoU multi-ancestry meta-analyses. Statistical test: two-sided maximum likelihood test statistic from logistic regression.

**Supplementary Table 5.** Full results of pathway enrichment analyses. Statistical test: two-sided overrepresentation test.

**Supplementary Table 6.** Full summary of the regression of HLA alleles on EBV DNAemia status. Statistical test: two-sided Wald test statistic from logistic regression.

**Supplementary Table 7.** Summary of predicted immunodominant epitopes per HLA allele with annotated IEDB validation.

**Supplementary Table 8.** Full annotation of population allele frequencies for EBV variants of unknown significance.
